# Getting Ready for Large-Scale Proteomics in Crop Plants

**DOI:** 10.3390/nu15030783

**Published:** 2023-02-03

**Authors:** Sarah Brajkovic, Nils Rugen, Carlos Agius, Nicola Berner, Stephan Eckert, Amirhossein Sakhteman, Claus Schwechheimer, Bernhard Kuster

**Affiliations:** 1 Chair of Proteomics and Bioanalytics, Technical University of Munich (TUM), 85354 Freising, Germany; 2 Institute of Plant Genetics, Leibniz University Hannover, 30167 Hannover, Germany; 3 Chair of Plant Systems Biology, Technical University of Munich (TUM), 85354 Freising, Germany

**Keywords:** plant proteomics, nutritional crop proteomics, liquid chromatography mass spectrometry

## Abstract

Plants are an indispensable cornerstone of sustainable global food supply. While immense progress has been made in decoding the genomes of crops in recent decades, the composition of their proteomes, the entirety of all expressed proteins of a species, is virtually unknown. In contrast to the model plant *Arabidopsis thaliana*, proteomic analyses of crop plants have often been hindered by the presence of extreme concentrations of secondary metabolites such as pigments, phenolic compounds, lipids, carbohydrates or terpenes. As a consequence, crop proteomic experiments have, thus far, required individually optimized protein extraction protocols to obtain samples of acceptable quality for downstream analysis by liquid chromatography tandem mass spectrometry (LC-MS/MS). In this article, we present a universal protein extraction protocol originally developed for gel-based experiments and combined it with an automated single-pot solid-phase-enhanced sample preparation (SP3) protocol on a liquid handling robot to prepare high-quality samples for proteomic analysis of crop plants. We also report an automated offline peptide separation protocol and optimized micro-LC-MS/MS conditions that enables the identification and quantification of ~10,000 proteins from plant tissue within 6 h of instrument time. We illustrate the utility of the workflow by analyzing the proteomes of mature tomato fruits to an unprecedented depth. The data demonstrate the robustness of the approach which we propose for use in upcoming large-scale projects that aim to map crop tissue proteomes.

## 1. Introduction

Plants constitute the nutritional basis of virtually all life on Earth, and protein-rich foods from crop plants are essential for sustaining an increasing human population and counteracting climate change. While the genomes of crops are increasingly elucidated, little is known about their proteomes. The proteome is the entirety of all proteins expressed by a plant. It is these proteins that execute and control nearly every aspect of that plant’s life. Proteomics is the large-scale study of proteomes and can examine the protein composition of a whole species such as tomato (*Solanum lycopersicum*) or just a single organ such as its fruit.

When compared to bacteria or animals, proteomic analyses of crop plants are often hindered by extreme concentrations of oxidative or proteolytic enzymes as well as often colorful secondary metabolites such as pigments, phenolic compounds, lipids, carbohydrates or terpenes. These are released from the plant material during protein extraction and often interfere with downstream processes such as protein solubilization, digestion into peptides or liquid chromatography coupled to tandem mass spectrometry (LC-MS/MS) [1]. Complicating matters further, the generally low protein concentration in most plant tissues often necessitates the use of large quantities of starting material. As a result, crop proteome projects have, so far, demanded individually optimized protein extraction protocols to obtain samples of acceptable quality [1].

Many published protocols for plant proteomics include a protein precipitation step prior to proteolysis. Traditional methods such as TCA/acetone or phenol extraction efficiently remove a wide range of compounds soluble in the organic solvent [2,3,4]. However, precipitated proteins can be hard to re-solubilize, leading to the loss of valuable sample material. Inconsistent precipitation and re-solubilization from sample to sample also compromises quantitative precision and accuracy. The issue of protein re-solubilization has been largely solved by the introduction of methods that support the use and subsequent removal of strong detergents or chaotropes [5,6].

Presumably because of the extreme molecular diversity of plant material and the many associated complications mentioned above, the plant proteomics community has relied on the use of two-dimensional gel electrophoresis (2-DE gel) in conjunction with mass spectrometry for protein identification and quantification for many years. This is in stark contrast to mainstream proteomics which rapidly replaced 2D gels by LC-MS/MS. The latter is by far superior to 2-DE gels in terms of proteome coverage, sensitivity, quantitative accuracy and the capacity to process and analyze large sets of samples [1,7]. The dominance of gel-based methods in the plant field has recently started to vane as the adaptation and evaluation of methods originally developed for animal samples also showed promising results for plants. Today, it is possible to use strong detergents (sodium dodecyl sulfate (SDS), sodium deoxycholate (SDC) or 3-((3-cholamidopropyl)dimethylammonio)-1-propanesulfonate (CHAPS)), chaotropes such as urea and thiourea or reducing agents such as dithiothreitol (DTT) for protein solubilization and denaturation from whole lysates. Combinations of these agents can further improve protein solubilization [8,9,10]. Another advantage is that one is no longer dependent on protein precipitation, which significantly reduces sample loss that can otherwise occur.

SDS is perhaps the best and most widely used protein solubilization agent. However, it also inhibits protease activity, is incompatible with certain forms of liquid chromatography and suppresses peptide ionization during MS analysis [11,12]. This necessitates the removal of SDS from a sample prior to protein digestion and LC-MS/MS analysis. Several methods have been developed over the past 10 years and are being increasingly adopted for plant samples. These include filter-aided sample preparation (FASP) [9,13,14,15] introduced in 2009, protein suspension trapping (S-Trap) reported in 2014 [16] and single-pot-solid-phase-enhanced sample preparation (SP3) published in 2019 [17,18].

FASP makes use of ultrafiltration columns that contain membranes with a 30 kDa molecular weight cut-off. When fully denatured, even small proteins have hydrodynamic volumes large enough to be retained while small organic molecules such as SDS are washed out during centrifugation. The proteins can then be directly digested on the membrane and peptides can be easily recovered for LC-MS/MS analysis [13]. That said, the FASP workflow can be rather time-consuming, and filter membranes have been known to develop blockages inexplicably, which poses a risk of complete sample loss. Full removal of SDS from FASP filters may also require an additional extraction step using ethyl acetate, thus increasing time demands and potential for sample losses [19].

A variation of the FASP idea is the protein suspension trapping (S-Trap) method. Here, proteins are solubilized with SDS and then acidified, leading to a suspension of protein precipitate in an aqueous buffer containing traces of SDS. This suspension is transferred into commercial S-Trap tips containing a filter to retain proteins and remove small molecules via centrifugation. Following protein digestion on the filter, peptides are retained by the hydrophobic component in the S-Traps, allowing direct peptide clean-up after digestion. As S-Traps are available in 96-well format and are compatible with different extraction buffers, they have become popular in mainstream proteomics [16,20,21].

In the recently introduced SP3 method, proteins are precipitated onto (paramagnetic) beads by the addition of high percentages of organic solvents, notably acetonitrile. SDS and other small molecules remain in the organic phase and are easily removed by washing beads with organic solvent. Digestion of proteins is performed directly on the beads, and prior work on single *Drosophila* embryos showed that SP3 can handle even minute amounts of protein [17,22]. The latter should facilitate the analysis of scarce plant materials such as pollen [23]. SP3 can also be automated in 96-well format on robotic platforms to increase throughput and reproducibility (termed AutoSP3) [24]. The method has been successfully applied to the analysis of *Arabidopsis* leaves and has the potential to become a standard approach for crop plants [18].

Results from two studies using leaf material from barley and *Arabidopsis* suggested that FASP is superior to the classic in-solution digest (ISD), but the study did not include the S-Trap or SP3 methods for comparison [9,10]. A recent paper comparing FASP, S-Trap and two different SP3 protocols using *Arabidopsis* leaves reported that S-Trap was inferior to FASP and SP3 with regard to the number of identified peptides and protein groups. In the same study, SP3 outperformed FASP for low sample input and gave comparable results for large sample amounts. Because SP3 is less time-consuming, cheaper and more easily automated, the authors projected that SP3 may become the preferred method for plant proteomics in the future [18].

Inevitably, the identification of peptides and proteins is performed by LC-MS/MS. For many years, the prevailing approach used nano-flow liquid chromatography coupled to electrospray ionization (ESI) tandem mass spectrometry. This is because of the superb sensitivity offered by this combination. However, the often extremely high concentrations of certain proteins in plant tissue, such as Rubisco in green tissues, degrades chromatographic performance and, in turn, quantitative precision. More recently, we have shown that chromatography operating at 50 μL/min (micro-flow LC-MS/MS) can alleviate this issue at a moderate cost of sensitivity [25,26]. We further showed that nearly 9000 proteins from Arabidopsis could be identified by such a system in a single 3-hour LC-MS/MS experiment [25,26].

The number of published comprehensive crop proteomes with relevance to human nutrition is still very small. The wheat (*Triticum aestivum*) proteome has been mapped to a depth of almost 16,000 proteins [27], that of maize to nearly 18,000 proteins [28] and there is a multitude of smaller-scale reports often on specific tissues, notably fruits. Tomato (*Solanum Lycopersicum*) is an interesting case as it is one of the most important crops worldwide with a global production nearing 200 million tons per year [29]. Tomato fruit is among the more challenging crop plant tissues to work with due to the presence of high amounts of sugars and secondary metabolites. Initial attempts to analyze the tomato fruit proteome led to the identification of a rather modest number of protein groups (85–1140, summarized in [30,31]). A substantial step forward was made by Kilambi et al., who reported the identification of 5404 proteins in seedless fruits following a multitude of sample preparation optimization steps and MS parameter adjustments [32]. Less than one year later, Szymanski et al. characterized the proteomes of tomato fruit skin and flesh at five time points during fruit development and reported the identification of 7738 proteins using a combination of FASP for protein digestion and high-pH reversed-phase liquid chromatography for peptide fractionation [30].

The authors of the present work have recently launched an initiative to map the proteomes of the 100 most important crop plants for human nutrition. This basic science project asks questions such as (i) which of the genes of a crop plant produce a protein product, (ii) where these proteins are expressed in the plant and (iii) in which approximate quantities. In order to realize this ambitious goal, it is necessary to reliably extract, prepare and analyze the proteins from all these different crops with their respective characteristics. In the current manuscript, we report on the development of an end-to-end workflow (from sample preparation to LC-MS/MS measurement, to protein identification and quantification) which enables us realize this project. All the elements have been published before, but combining them in the way described enables high-throughput, high quality and high proteomic coverage at the same time. The merits of the workflow are exemplified by the analysis of whole tomato fruits leading to the identification of 140,000 peptides and 9900 proteins with a median quantitative precision of <10% coefficient of variation (CV).

## 2. Materials and Methods

### 2.1. Plant Material and Growth Conditions

*Solanum lycopersicum* cv. M82 plants were grown in 15 L pots on peat substrate (C700, Stender GmbH, Schermbeck, Germany). Twelve weeks after planting, 3 tomato fruits from each of the 3 plants were harvested and freeze-dried in an ALPHA 1-2 LDplus (Christ Martin™, Osterode am Harz, Germany). The harvested tomato fruits were at the stage of red ripening. The harvested tomato fruits were ground in a TissueLyser II (QIAGEN, Hilden, Germany) in two cycles of 2 min grinding. Fine plant powder was stored at −80 °C.

### 2.2. Total Protein Extraction

Proteins were extracted from tomato fruit using the trichloroacetic acid/acetone precipitation with the phenol extraction method described earlier [1].

Briefly, finely powdered tomato fruits were subjected to metabolite extraction and protein precipitation with 2 mL of pre-chilled 10% trichloroacetic acid (*v*/*v*) in acetone. After overnight incubation at 4 °C, samples were centrifuged (15,000× *g*, 5 min, 4 °C). The pellet was washed three times with pre-chilled acetone, then air dried and resuspended in 1 mL of SDS extraction buffer (4% SDS, 150 mM Tris-Cl, pH 8.8, 1 mM EDTA, and 2 mM phenylmethylsulfonyl fluoride (PMSF)), followed by incubation at 60 °C for up to 1 h until the pellet had completely dissolved. Samples were then centrifuged to remove cell debris (15,000× *g*, 10 min, RT). An equal volume of Tris-saturated phenol (pH 7.5–8) was added to the supernatant and vortexed for 1 min. The phenolic phase was separated by centrifugation (15,000× *g*, 5 min, RT). The proteins were precipitated overnight at −20 °C with 1 mL of 0.1 M ammonium acetate in methanol. Protein pellets were obtained by centrifugation (15,000× *g*, 10 min, 4 °C). The pellets were washed once with 0.1 M ammonium acetate in methanol and twice with precooled 80% acetone (*v*/*v*) and then air dried. Finally, proteins were re-solubilized in 250 μL SDS-containing lysis buffer (4% SDS, 40 mM Tris-Cl, pH 7.6), followed by sonication using an R230 focused-ultrasound instrument (Covaris Ltd., Brighton, UK, 300 s duration, 30 s on/off) and a final centrifugation step (21,000× *g*, 60 min, 4 °C). Proteins were quantified using a bicinchoninic acid assay (BCA, Thermo Pierce).

### 2.3. SP3 Sample Preparation and Tryptic Digestion

A total of 200 μg of protein lysate from each tissue was processed by protein aggregation capture on a Bravo Agilent pipetting system using Sera-Mag^TM^ Carboxylate-Modified Magnetic Beads (Cytiva Europe GmbH, Freiburg im Breisgau, Germany) as previously described [17,18].

Briefly, 200 μg of lysate was mixed with washed Sera-Mag^TM^ magnetic beads (1:1 mixture of A and B, Cytiva) at a ratio of 1:5 (protein:bead). Proteins were precipitated by adding ethanol to a final concentration of 70%. The supernatant containing non-protein compounds was removed by capturing the beads by a magnet. The beads were washed three times with 80% ethanol. Finally, the beads were washed with 100% acetonitrile to remove any residual ethanol. Proteins were reduced and alkylated in 100 μL reduction and alkylation buffer (200 mM EPPS, pH 8.5, 55 mM CAA, 10 mM TCEP) for 1 h at 37 °C. Overnight enzymatic digestion was carried out using 5 µg trypsin at 37 °C. The tryptic digest was acidified with formic acid and desalted using CHROMABOND HLB desalting plates (10 mg N-Vinylpyrrolidon-Divinylbenzol porous particles 30 μm, MACHEREY-NAGEL). Peptides were eluted with 200 µL 70% acetonitrile and 0.1% formic acid and dried down in a speed-vac and stored at −20 °C until further use.

### 2.4. Peptide Fractionation

Peptide concentration was determined using a NanoDrop™ UV spectrophotometer (Thermo Scientific™, Waltham, MA, USA). A total of 100 μg of peptides was fractionated by basic pH reversed-phase material (RPS cartridge tips; 5 μL PS-DVB resin, Agilent, Santa Clara, CA, USA) into six fractions using the Agilent AssayMAP Bravo pipetting system. The RPS cartridges were primed, washed and equilibrated according to the manufacturer’s protocol. Peptides were reconstituted in 100 μL of 25 mM ammonium formate (pH 10) and loaded onto the cartridges. Peptides were fractionated by increasing acetonitrile concentrations (5%, 10%, 15%, 20%, 25%, 30%, 80%). The seven elution steps were combined into 6 fractions, combining the 5% and 80% fractions. All fractions were acidified with formic acid to a final concentration of 1%. The fractionated peptides were dried down in the speed-vac and stored at −20 °C until MS measurement. Before analysis by LC-MS, peptides were dissolved in 0.1% formic acid and were spiked with retention time standard peptides PROCAL [33] at 100 fmol per injection.

### 2.5. Mass Spectrometry

Micro-flow liquid chromatography tandem mass spectrometry was performed on a Vanquish Neo UHPLC system (Thermo Fisher Scientific) coupled online to an Orbitrap Eclipse Tribrid mass spectrometer (Thermo Fisher Scientific) operating in positive ion mode as previously described [25]. Briefly, samples were loaded directly onto the Acclaim PepMap 100 C18 column (2 μm particle size, 1 mm ID × 150 mm). The peptide mixture was separated at a flow rate of 50 μL/min using a linear gradient of acetonitrile from 3 to 28% (*v*/*v*), formic acid 0.1% (*v*/*v*) and 3% (*v*/*v*) DMSO and at a column temperature of 55 °C for 60 min. The eluting peptides were directly sprayed into the heated electrospray ionization (HESI) source of the mass spectrometer. Tandem mass spectra were acquired in DDA mode. From each MS scan, precursors were targeted for MS/MS scans if the charge was between 2 and 6 and the intensity exceeded 1e4. Fragmentation of the peptides was performed by higher-energy collision-induced dissociation (HCD).

### 2.6. Peptide and Protein Identification

MaxQuant (version 2.0.1.0) [34] with its built-in search engine Andromeda was used for peptide and protein identification and quantifications. MS/MS spectra were searched against the Uniprot tomato sequence database (34,658 entries; downloaded on 26 July 2022) as well as against the ITAG 4.0 database (34,075 entries, https://solgenomics.net/; downloaded on 26 July 2022) [35]. For MaxQuant, iBAQ and LFQ were used. Unless otherwise specified, the default parameters of MaxQuant were used. Trypsin/P was chosen as the proteolytic enzyme with up to two allowed missed cleavages. Carbamidomethylation of cysteine was chosen as a fixed modification whereas N-terminal protein acetylation and oxidation of methionine residues were chosen as variable modifications. The false discovery rate (FDR) for peptide spectrum matches (PSMs) and proteins was determined using a target-decoy approach with reversed protein sequences. The MaxQuant search was performed either with filtering for 1% FDR at the PSM level and without (100%) filtering of FDR at the PSM level. The 100% FDR MaxQuant search results were re-scored using the deep neural network Prosit [36].

The proteomic data (raw MS files and MaxQuant result files) have been deposited to the ProteomeXchange Consortium via the PRIDE partner repository with the dataset identifier PXD038945 [37].

## 3. Results

### 3.1. End-to-End-Workflow

**Figure 1:**
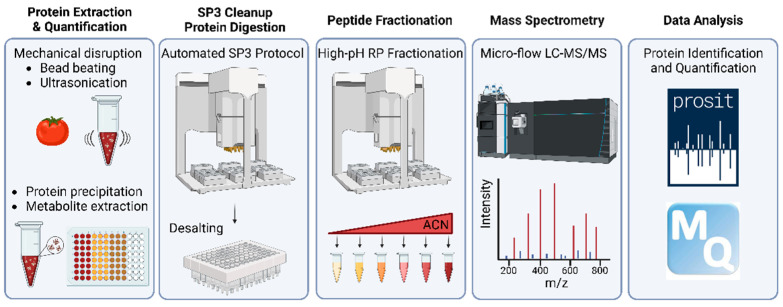
Schematic representation of the plant crop proteome profiling workflow developed in this study. ACN: acetonitrile.

The end-to-end workflow presented in this study is depicted in Figure 1. It comprises five main steps briefly described here (see Section 2 for details). In the first step (taking approximately three days of work for 96 samples), plant material is mechanically disrupted by bead beating and sonication, which also shears DNA. Then, proteins are precipitated by 10% TCA/acetone, recovered by SDS and precipitated again by phenol. The two rounds of protein precipitation and organic solvent extraction effectively remove all non-proteinous material. In step two (one day of work), proteins are again recovered in SDS, precipitated onto SP3 beads by ethanol (in 96-well format), on-bead digested using trypsin and the resulting protein digest is desalted using hydrophobic solid-phase extraction cartridges. In step three (one day of work), the digest is partially separated by high pH reversed-phase chromatography into six fractions. In step four, each fraction is analyzed by 60 min online micro-flow LC-MS/MS (four days of instrument time), and in step five, proteins are identified using MaxQuant/Andromeda with Prosit re-scoring and quantified by MaxQuant (two days of data handling and computation time).

### 3.2. Experiment Design

**Figure 2:**
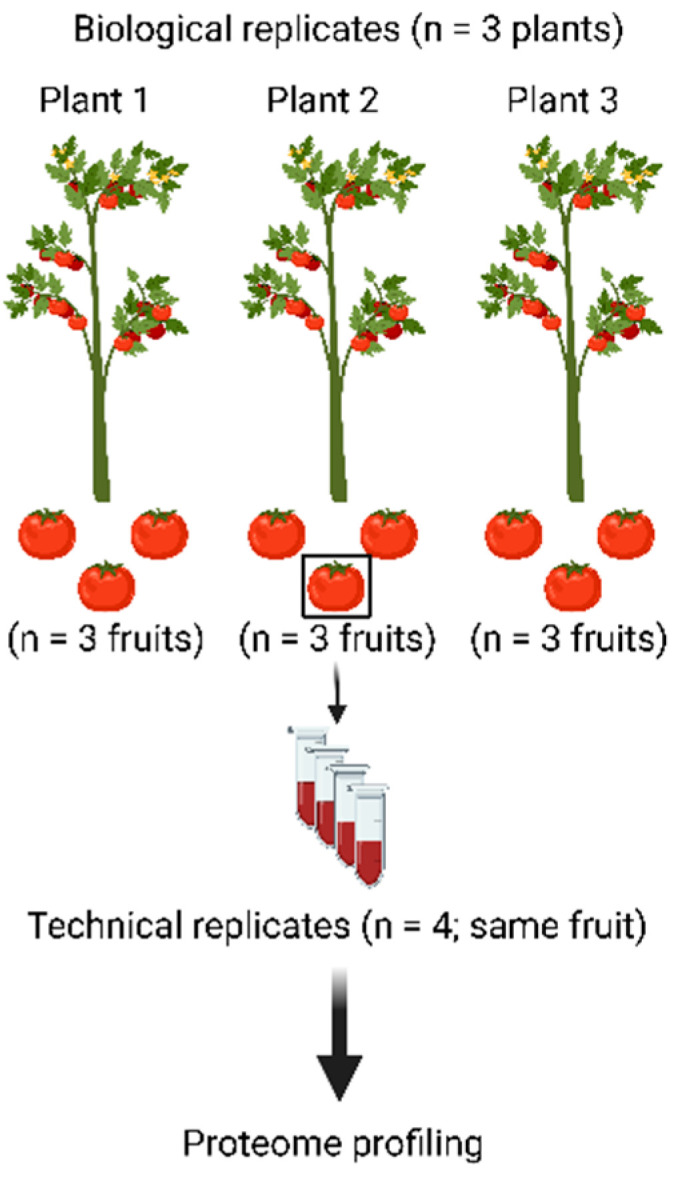
Experimental design to assess the merits of the proteomic workflow. Nine tomato fruits were harvested from three different plants and all were processed using the workflow depicted in Figure 1. This way, the biological variation from fruit to fruit and from plant to plant can be separated from the technical variation of the workflow, which was assessed by subjecting one tomato fruit to the complete workflow in four technical replicates.

To assess the merits of the workflow presented above, three tomato plants were grown and three fruits were harvested from each plant (Figure 2). All nine fruits were processed as described above to be able to assess plant-to-plant variation as well as fruit-to-fruit variation. In addition, material from a single fruit was processed four times (starting from powder) to assess the technical variation of the workflow.

### 3.3. Peptide and Protein Identification

**Figure 3:**
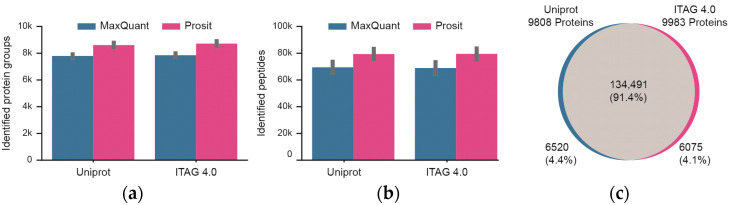
Summary of peptide and protein identifications of technical workflow replicates (*n* = 4). (**a**) Identification of proteins when searching the Uniprot or TAG 4.0 sequence databases and either including or excluding re-scoring of MaxQuant results using the artificial intelligence Prosit. Black bars represent one standard deviation. (**b**) Same as panel (**a**) but for peptides. (**c**) Venn diagram showing the overlap of peptides identified from Uniprot or ITAG 4.0.

The mass spectrometric data were searched against two tomato protein sequence databases (Uniprot, ITAG 4.0) that contain a similar, but not identical number of protein sequences. In addition, the database search results were processed and either included or excluded Prosit re-scoring (Figure 3; Tables S1 and S2). Focusing first on the four technical workflow replicates, the number of identified peptides and proteins between replicates was highly consistent. For Uniprot, a median of 8686 +/− 210 stdev proteins and 81,107 +/− 4482 stdev peptides were identified. The respective figures for the ITAG 4.0 database are 8795 +/− 214 stdev proteins and 81,365 +/− 4719 stdev peptides. Prosit re-scoring of Uniprot or ITAG 4.0 database search results led to a median increase of 15% in peptide and 11% in protein identifications over MaxQuant alone. Taking all of the data together and including Prosit rescoring, a total of ~147,000 peptides and ~9900 proteins were identified at a false discovery rate of 1%. The overlap between the two database search results was also very high (>91% at the peptide level). The overlap at the protein level could not be assessed because the Uniprot and ITAG 4.0 protein identifiers are not the same and no mapping table was available. Still, the fact that only ~4% of the identified peptides were unique to either Uniprot or ITAG 4.0 implies that the protein sequence content of the two databases are similar. Because slightly more proteins were identified from the ITAG 4.0 database, all further data analysis is based on this sequence collection.

### 3.4. Quantitative Precision of the Workflow and Biological Proteome Variation

**Figure 4:**
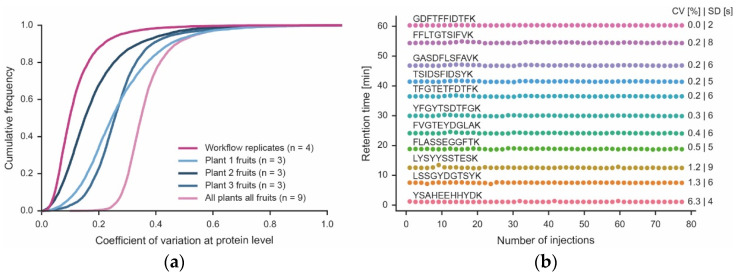
Quantitative precision of the workflow and biological proteome variation. (**a**) Cumulative density plot of the quantitative precision (expressed as coefficient of variation, CV) of four technical workflow replicates using material from the same tomato fruit, three different fruits of each of three plants (Plant 1–3) and all tomato fruits from all plants combined. (**b**) Retention time stability of 12 synthetic peptides spiked into each of the 78 samples (injections) analyzed in this study. Each sample was analyzed by a 60 min LC-MS/MS run. The retention time variation of each peptide is expressed as CV and standard deviation (SD).

Analysis of the four technical workflow replicates showed that the data only needed minimal normalization (Figure S4). In addition, the peptide intensities provided by the MS measurement showed Pearson correlation coefficients of 0.99 between any replicate (Figure S5). More specifically, the median quantitative precision was <10% CV, and ~90% of all proteins were within 20% CV (Figure 4). As one would expect, CVs were larger for low-abundance proteins than for higher-abundance proteins (Figure S3). Achieving such low CVs was, in part, facilitated by the high reproducibility of the micro-flow chromatographic system. It is apparent from Figure 4 that the retention times of the peptide standards that were spiked into every one of the 78 samples analyzed here (representing all fractions of all technical and biological replicates) was stable (median CV of 0.4% (*n* = 12 monitored peptides). Surprisingly, there was considerable variation between three fruits of the same plant (median CVs of 15–25%; 90% of all proteins within 35–45% CV) and even more variation between fruits of different plants (median CV of 35; 90% of all proteins within 45% CV). Given the low technical variation in the workflow, protein expression changes greater than 2-fold (5x CV for 90% of all proteins) should be confidently biologically interpretable.

### 3.5. Proteome Coverage and Workflow Bias

**Figure 5:**
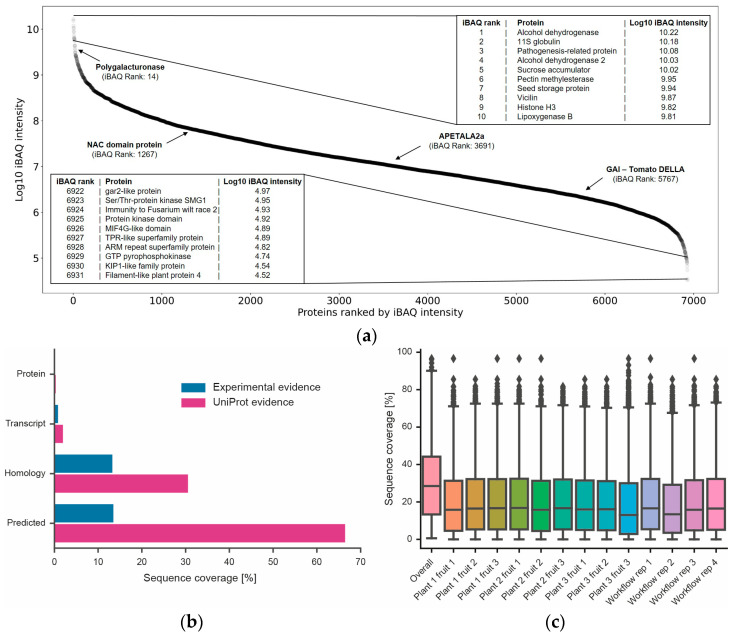
Dynamic range of proteome expression, discovery of new proteins and amino acid sequence coverage. (**a**) Ranked protein abundance plot and gene names of the 10 most highly expressed proteins and 10 least highly expressed proteins covered by the data. Four proteins important for tomato fruit development are also highlighted [38]. (**b**) Percentage of all tomato genes divided by difference evidence levels according to UniProt (orange). Blue bars represent proteins identified in this study. (**c**) Box plots showing the distribution of amino acid sequence coverage for proteins identified in each experiment or when combining all data into one analysis.

With the identification of ~9900 proteins, the current study represents the most comprehensive tomato proteome published to date and provides evidence for the existence of a protein product for many genes for the first time (Figure 5). The dynamic range of protein expression spanned six orders of magnitude (Figure 5), suggesting that the tomato fruit proteome is reasonably comprehensive. The data do not only cover relatively high-abundance proteins, but also include kinases and transcription factors often presumed to be of low cellular abundance. In line with general experience, the amino acid sequence coverage of all proteins rarely exceeded 30% in a single sample and seldom 40% when combining all data.

**Figure 6:**
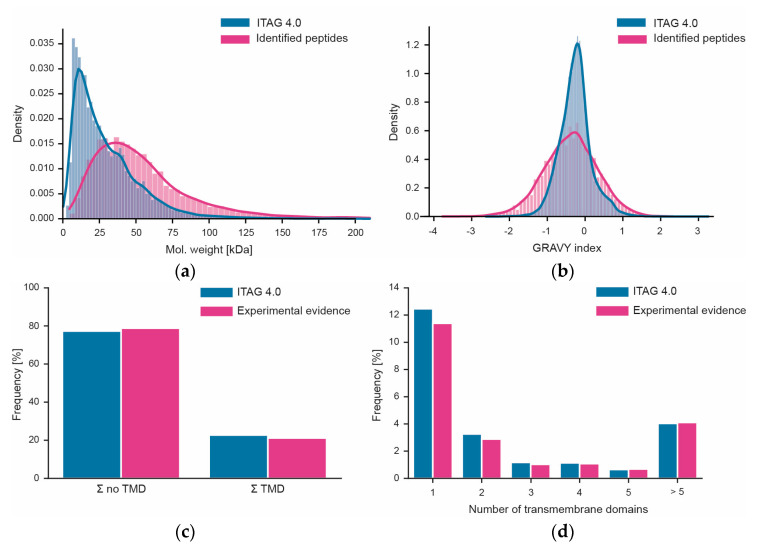
Assessment of workflow bias. (**a**) Distribution of the molecular weights of all proteins in ITAG 4.0 vs. proteins identified in this study; (**b**) same as panel (**a**) but for peptide hydrophobicity (expressed as GRAVY index). (**c**) Fraction of proteins with or without predicted transmembrane domains (TMF) in ITAG 4.0 or identified in this study. (**d**) Percentage of proteins with different numbers of predicted transmembrane domains in ITAG 4.0 or identified in this study.

We also attempted to assess to what extent the workflow used here may be biased in terms of which proteins can or cannot be covered. Unfortunately, performing systematic gene ontology (GO) or gene set analysis could not be performed as only about 17% of all tomato proteins in ITAG 4.0 have GO annotations. Instead, we looked at simpler biochemical parameters such as the molecular weight distribution of proteins. Superimposing the distributions of proteins from the sequence database and the proteins identified in this study showed that proteins of all sizes can be covered by the workflow (Figure 6). However, there was a strong underrepresentation of proteins smaller than 20 kDa. In contrast, the distribution of the hydrophobicities of tryptic peptides measured in this work covered >99% of the range of peptide hydrophobicities in ITAG 4.0 showing that there is no bias at the level of peptides. This is important because the workflow measures peptides, not proteins (also see discussion section). There is also no bias in the data in terms of the coverage of transmembrane domain-containing proteins (Figure 6). This was true overall, but also when breaking down the data by the number of membrane-spanning domains.

## 4. Discussion

The workflow presented in this study was designed to support a large number of samples from which we intend to build a crop proteome atlas covering the 100 most important crop plants for human nutrition. None of the elements in the workflow are new as such. However, the way they were put together is novel and has a few interesting features.

For instance, the workflow contains three steps in which small molecule components are extracted by organic solvent. First during TCA/acetone precipitation, second during phenol extraction and third during ethanol precipitation onto SP3 beads. This generates protein preparations that are essentially free of non-proteinous material, which greatly contributes to the robustness of the workflow. This comes at the price of losing about 50% of the total protein content of a sample. Considering that most crop plant samples are abundantly available and the fact that the workflow only requires a total amount of 200 ug of protein, the loss of material is a lesser concern. In addition, most steps are performed in 96-well format, enabling a high degree of parallelization and automation. This not only increases sample throughput but, more importantly, improves reproducibility. The end-to-end technical variation of the workflow is very low as quantitative precision is better than 10% CV for half of all proteins and better than 20% for 90% of all proteins. These figures of merit were partly achieved by operating the chromatography part of the LC-MS/MS system at microliter flow rates rather than the more typical nanoliter flow rates. This renders the chromatography more robust and reduces overhead times [25,26,39]. The bottleneck of the workflow is the time needed for LC-MS/MS analysis (6 h per proteome). Still, in the current configuration, up to 28 crop proteome samples may be collected within one week, albeit 24 is more realistic considering that instrumentation also requires maintenance.

Nearly 10,000 proteins were identified from tomato fruit in this study, rendering it the largest tomato proteome published to date. These proteins span nearly six orders of magnitude of dynamic range. In other words, it is possible to detect proteins that are present with only 10 copies per cell in the presence of other proteins with >10 million copies per cell. In this regard, the workflow should cover the proteome of the fruit rather comprehensively. Still, the tomato fruit data only cover 30% of the total proteome as estimated from the prediction of coding regions from the tomato genome. This is substantially lower than what our laboratory achieved when mapping the proteomes of Arabidopsis (66% coverage), mouse (76% coverage) and human (68% coverage) [23,40,41]. However, these studies analyzed many tissues, each of which also only covered about 10,000 proteins. Hence, it is reasonable to expect that a more substantial increase in the coverage of the tomato proteome will come from analyzing other tissues where other proteins are more abundant. In addition, the strong bias we observed against the detection of low-molecular-weight proteins raises the question if ITAG 4.0 contains rather too many small proteins, as these are notoriously difficult to call correctly by all major gene prediction programs [42].

This study did not include the analysis of post-translational modifications, as this is not the current focus of the crop proteome atlas project. However, the presented workflow can be easily adapted to include the enrichment of phosphorylated peptides. This could be achieved by depleting phosphopeptides via immobilized metal affinity chromatography (IMAC) from the same sample prior to peptide separation by high pH reversed phase. The AssayMap Bravo platform supports IMAC consumables in 96-well format, which would not add a lot of sample processing time but considerable additional LC-MS/MS time would be required for including a phosphoproteome analysis.

In conclusion, the proteomic workflow presented here should be fit for its main purpose, which is measuring proteome expression in crop plants at a large scale. It can also be deployed to support projects of different size and scope, which is why it can be anticipated that the workflow will be readily taken up by others, especially in the field of plant research.

## 5. Conclusions

The proteomic workflow presented here should be fit for its main purpose, which is measuring proteome expression in crop plants at a large scale. It scales to cover a crop plant proteome to the depth of about 10,000 proteins and in a quantitative fashion. It can also be deployed to support projects of different size and scope, which is why it can be anticipated that the workflow will be readily taken up by others, especially in the plant research community.

## Data Availability

All raw mass spectrometry data as well as MaxQuant output files have been deposited with the proteomeXchange consortium via the partner repository PRIDE. Data are available using the accession PXD038945.

## Supplementary Materials

The following supporting information can be downloaded at: https://www.mdpi.com/article/10.3390/nu15030783/s1, Figure S1: Cumulative density plot described by the coefficient of variation based on peptide LFQ intensity; Figure S2: Cumulative protein abundance; Figure S3: Log10 iBAQ intensity against coefficient of variation; Figure S4: Boxplots of the non- and median-normalized workflow replicates; Figure S5: Correlation plot of workflow replicates; Table S1: Number of identified protein groups and peptides using MaxQuant; Table S2: Number of identified protein groups and peptides using Prosit.
