# Supplementary material for: Getting Ready for Large-Scale Proteomics in Crop Plants
Source: Nutrients. 2023 Feb 3;15(3):783. doi: 10.3390/nu15030783 (PMC9921824; doi:10.3390/nu15030783)
Supplement: Supplementary file 1 [file nutrients-15-00783-s001.zip › nutrients-2174487-supplementary.pdf]

Supplementary Materials

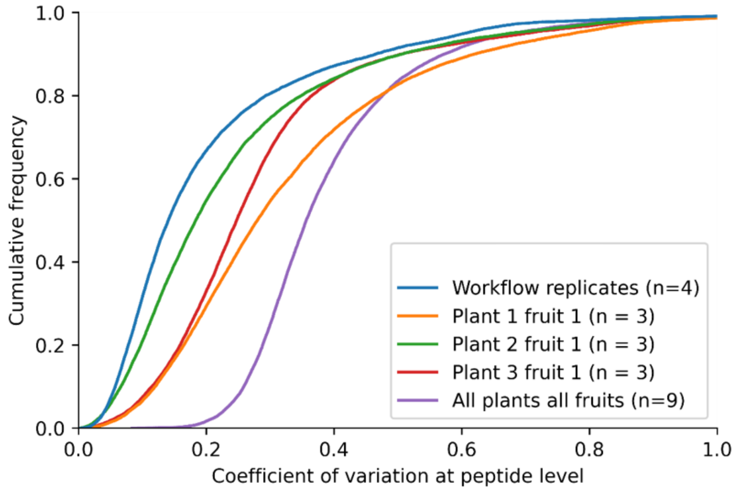

Figure S1. Cumulative density plot showing the coefficient of variation based on peptide LFQ intensities.

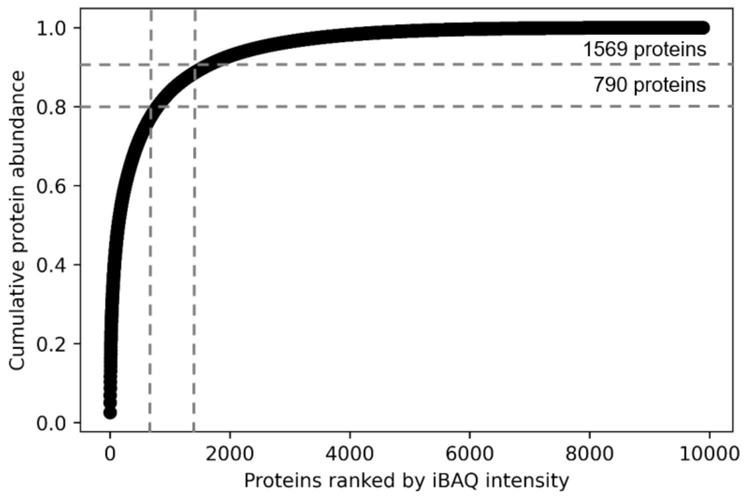

Figure S2. Cumulative protein abundance ranked by iBAQ protein intensity. Each black dot represents one protein.

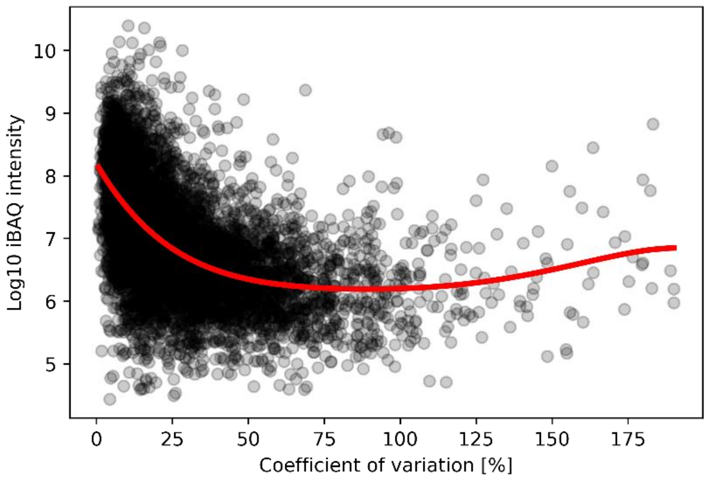

Figure S3. Coefficient of variation of technical workflow replicates (n=4) as a function of protein abundance (expressed as log10 iBAQ intensity). Each black dot represents one protein. Red line represents the 5th order polynomial trendline.

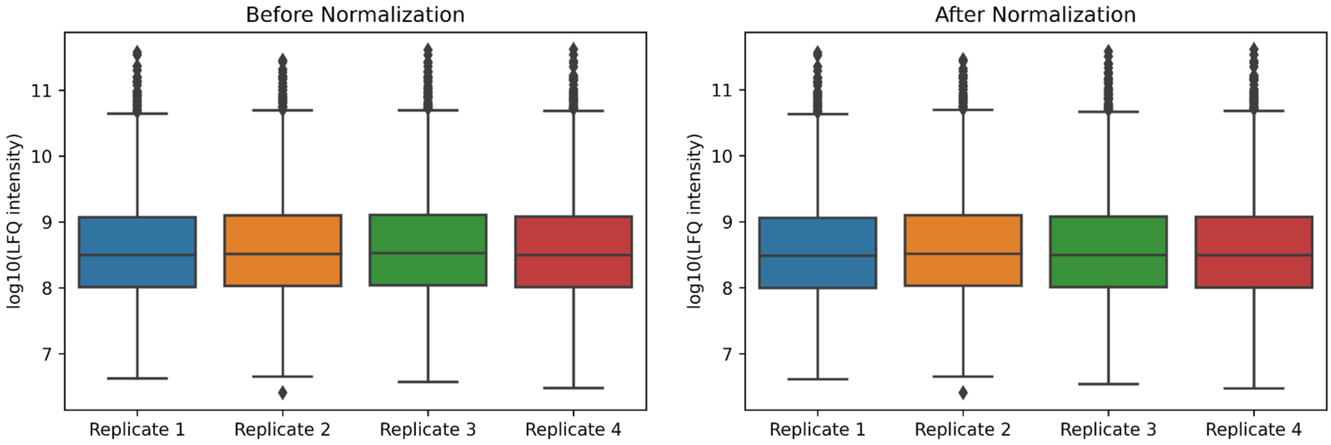

Figure S4. Boxplots of the LFQ protein intensities before (left panel) and after median-normalization (right panel). Data from workflow replicates.

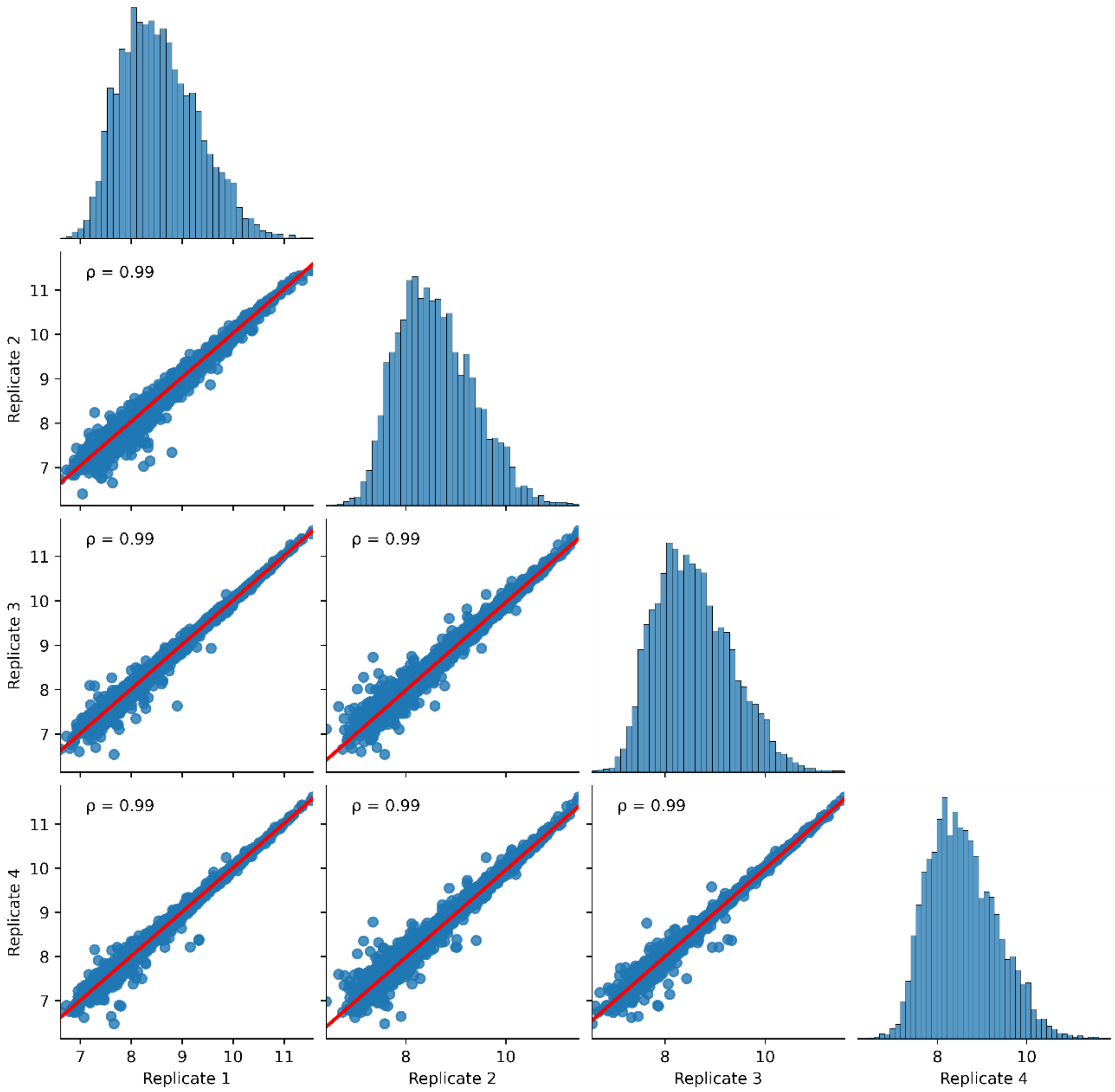

Figure S5. iBAQ protein intensity distributions and pair-wise correlation plots of iBAQ protein intensities of the four workflow replicates. p = Pearson correlation coefficient.

Table S1. Number of identified protein groups and peptides using MaxQuant.

**MaxQuant**	**# Protein Groups (Uniprot)**	**# Peptides (Uniprot)**	**# Protein Groups (ITAG 4.0)**	**# Peptides (ITAG 4.0)**
Plant 1	7671	68278	7757	67915
	7891	72047	7931	71571
	7787	64848	7915	69633
Plant 2	7788	69961	7860	69382
	7720	67833	7789	67085
	7871	72423	7907	71128
Plant 3	7669	67854	7703	67482
	7732	68955	7767	68004
	7369	68309	7473	70675
Workflow Replicates	7892	71032	7952	70676
	7584	62591	7603	61590

	7792	70193	7874	69998
	7891	73884	7957	73372
Total Proteome	8270	117607	8298	114937

Table S2. Number of identified protein groups and peptides using Prosit re-scoring of MaxQuant results.

**Prosit**	# **Protein Groups (Uniprot)**	# **Peptides (Uniprot)**	# **Protein Groups (ITAG 4.0)**	# **Peptides (ITAG 4.0)**
Plant 1	8517	80321	8651	80285
	8808	82411	8897	82434
	8700	76622	8884	81499
Plant 2	8718	81076	8833	81271
	8574	78022	8650	77874
	8768	82706	8909	82468
Plant 3	8624	80130	8762	80787
	8572	79891	8696	79778
	8027	78238	8156	81267
Workflow Replicates	8755	81608	8875	81933
	8295	72715	8402	72478
	8665	80606	8777	80796
	8707	82443	8812	82648
Total Proteome	9808	141011	9938	140566
